# Dickkopf-3 Causes Neuroprotection by Inducing Vascular Endothelial Growth Factor

**DOI:** 10.3389/fncel.2018.00292

**Published:** 2018-09-11

**Authors:** Carla Letizia Busceti, Luisa Di Menna, Franca Bianchi, Federica Mastroiacovo, Paola Di Pietro, Anna Traficante, Giovanna Bozza, Christof Niehrs, Giuseppe Battaglia, Valeria Bruno, Francesco Fornai, Massimo Volpe, Speranza Rubattu, Ferdinando Nicoletti

**Affiliations:** ^1^IRCCS Neuromed, Pozzilli, Italy; ^2^Division of Molecular Embryology, DKFZ-ZMBH Allianz, German Cancer Research Center, Heidelberg, Germany; ^3^Institute of Molecular Biology (IMB), Mainz, Germany; ^4^Department of Physiology and Pharmacology, University Sapienza, Rome, Italy; ^5^Department of Human Morphology and Applied Biology, University of Pisa, Pisa, Italy; ^6^Clinical and Molecular Medicine, University Sapienza, Rome, Italy

**Keywords:** Dickkopf-3, VEGF, neurodegeneration, cerebral ischemia, astrocytes

## Abstract

Dickkopf-3 (Dkk3) is an atypical member of the Dkk family of Wnt inhibitors, which has been implicated in the pathophysiology of neurodegenerative disorders. However, the role of Dkk3 in mechanisms of cell degeneration and protection is unknown. We used Dkk3 knockout mice to examine how endogenous Dkk3 influences ischemic brain damage. In addition, we used primary cultures of astrocytes or mixed cultures of astrocytes and neurons to investigate the action of Dkk3 on cell damage and dissect the underlying molecular mechanisms. In a model of focal brain ischemia induced by permanent middle cerebral artery (MCA) occlusion (MCAO) Dkk3^−/−^ mice showed a significantly greater infarct size with respect to their wild-type counterparts at all time points investigated (1, 3 and 7 days after MCAO). Immunohistochemical analysis showed that Dkk3 expression was enhanced at the borders of the ischemic focus, and was predominantly detected in astrocytes. This raised the possibility that Dkk3 produced by astrocytes acted as a protective molecule. We tested this hypothesis using either primary cultures of cortical astrocytes or mixed cortical cultures containing both neurons and astrocytes. Genetic deletion of Dkk3 was permissive to astrocyte damage induced by either oxidative stress or glucose deprivation. In addition, application of human recombinant Dkk3 (hrDkk3) was highly protective against oxidative stress in cultured astrocytes. We tested the hypothesis that the protective activity of Dkk3 was mediated byvascular endothelial growth factor (VEGF). Interestingly, glucose deprivation up-regulated both Dkk3 and VEGF in cultured astrocytes prepared from wild-type mice. VEGF induction was not observed in astrocytes lacking Dkk3 (i.e., in cultures prepared from Dkk3^−/−^ mice). In mixed cultures of cortical cells, excitotoxic neuronal death induced by a brief pulse with *N*-methyl-D-aspartate (NMDA) was significantly enhanced when Dkk3 was lacking in astrocytes, whereas post-NMDA addition of hrDkk3 was neuroprotective. Neuroprotection by hrDkk3 was significantly reduced by pharmacological blockade of type-2 VEGF receptors and was mimicked by hrVEGF. These data offer the first evidence that Dkk3 protects both neurons and astrocytes against a variety of toxic insults, and at least in culture, protection involves VEGF induction.

## Introduction

The Dickkopf (Dkk) family of Wnt inhibitors includes four secreted proteins (Dkk-1 to -4), of which Dkk3 diverges from the other members for the presence of a soggy-like domain in addition to common DKK_N and colipase fold domains (Kawano and Kypta, 2003). As opposed to Dkk-1, the inhibitory action of Dkk3 on the Wnt pathway is context-dependent and relies on the expression levels of the Wnt co-receptors, LRP5 and -6 (Caricasole et al., 2003). A large body of evidence suggests that Dkk3 is a key regulator of basic cellular processes, such as cell proliferation, differentiation and apoptotic death. The role of Dkk3 in mechanisms of cell death and survival is also context-dependent. Dkk3 behaves as an oncosuppressor protein by inducing apoptotic death in a variety of cancers (Hoang et al., 2004; Hsieh et al., 2004; Ueno et al., 2011; Dellinger et al., 2012; Veeck and Dahl, 2012; Yang et al., 2012; Eskander et al., 2016; Lorsy et al., 2016; Sawahara et al., 2016), although it might favor cancer cell spreading by promoting angiogenesis (Untergasser et al., 2008; Zitt et al., 2008). Dkk3 induces kidney tubular cell death in proteinuric nephrosis (Wong et al., 2016), but, in contrast, it supports cell survival in the mouse retina (Nakamura et al., 2007). The following observations raised our interest on the involvement of Dkk3 in mechanisms of neurodegeneration/neuroprotection in the CNS: (i) Dkk3 co-localizes with β-amyloid peptide both in diffuse and classic plaques in brain tissue from patients affected by Alzheimer’s disease (AD; Bruggink et al., 2015); (ii) Dkk3 levels are reduced in brain tissue from AD patients and transgenic mouse models of AD (Zhang et al., 2017); (iii) brain-specific enhancement of Dkk3 expression in AD mice improves amyloid pathology, cognitive dysfunction and cerebral glucose metabolism (Zhang et al., 2017); and (iv) Dkk3 induces the expression of vascular endothelial growth factor (VEGF) in cultured endothelial cells (Busceti et al., 2017) and VEGF was shown to exert protective activity against hypoxic/ischemic and excitotoxic neuronal injury (Svensson et al., 2002; Moser and Humpel, 2005; Taoufik et al., 2008; Inada et al., 2014).

These observations laid the groundwork for the present study aimed at establishing whether Dkk3 affects the development of cell death in cultured astrocytes or neurons exposed to different insults, and whether genetic deletion of Dkk3 shapes ischemic vulnerability *in vivo*.

## Materials and Methods

### Materials

Human recombinant Dkk3 and VEGF (hrDkk3, hrVEGF) were purchased from RD System, Minneapolis, MN, USA (codes: 1118-DK and 293-VE-500, respectively). ZM 3238881 and *N*-methyl-D-aspartate (NMDA) were purchased from Tocris Bioscience, Bristol UK, code: 2475.

### Animals

CD1 mice (Charles River, Italy) were used for the preparation of pure astrocyte or mixed cortical cultures, and for the analysis of Dkk3 and VEGF levels in the whole brain. Dkk3^−/−^ mice were kindly provided by CN. Dkk3^−/−^ mice and their wild-type littermates (C57BL/6J strain) generated by heterozygous breeding were used for cell culture studies. For *in vivo* studies, wild-type and Dkk3^−/−^ mice were generated by heterozygous breeding. All animals were housed under controlled conditions on a 12 h light-dark cycle with food and water *ad libitum*. Experimental protocols used for the study were approved by the Neuromed Institute Ethical Committee and subsequently approved by the Italian Ministry of Health (Authorization #: 731/2017-PR).

### Dkk3 Knockout Mouse Genotyping

Genotyping of Dkk3^−/−^ mice was performed by a triplex PCR on genomic tail DNA using the following oligonucleotides: 5-GATAGCTTTCCGGGACACAC-3, 5-TCCATCAGCTCCTCCACCTCT-3, and 5-TAAGTTGGGTAACGCCAGGGT-3 to produce 220-bp and 199-bp bands from the wild-type and targeted allele, respectively.

### Mouse Model of Permanent Focal Ischemia

Ten week-old wild-type or Dkk3^−/−^ male mice were used for surgical induction of permanent focal ischemia. Mice were anesthetized with chloral hydrate (320 mg/kg, i.p.) and an incision was made between the outer canthus of the eye and the external auditory meatus. The temporal muscle was bisected and the middle cerebral artery (MCA) was exposed by means of a burr-hole craniotomy carried out by a surgical drill. The MCA was occluded by electrocoagulation (Tamura et al., 1981; Mastroiacovo et al., 2009). The temporal muscle and then the skin incision were sutured using 5/0 sutures. During surgery body temperature was maintained at 37°C with rectal probe connected to a heating pad. After surgery, all mice were placed in a thermal incubator at 37°C for 2 h (Compact Incubator, Thermo Scientific, AHSI, Italy). For sham operation, animals were subjected to the same anesthesia and surgical procedures, except for MCA occlusion (MCAO). Animals were killed at 1, 3 and 7 days after MCAO, and were used for histological/immunohistochemical analysis.

### Histological Analysis

Brains of mice were fixed in Carnoy’s solution and embedded in paraffin. The sections of 10 μm regularly spaced every 550 μm through the extension of the ischemic region were deparaffinized and processed for staining with thionin (Nissl staining for histological assessment of neuronal degeneration). The necrotic area was outlined at a magnification of 2.5× and it was quantified using Scion Image software (NIH, Bethesda, MD, USA). The extent of the infarct volume was calculated by integrating the cross-sectional area of damage at each stained section and the distances between the various sections (Osborne et al., 1987).

### Immunohistochemical Analysis

At different times following MCAO, C57BL/6J mice were transcardially perfused with paraformaldehyde (PFA, 4% in 0.1 M phosphate buffer, pH 8). Dissected brains were post-fixed in 4% PFA (+4°C, overnight), cryo-protected in 30% sucrose and cut at cryostat in slices of 30 μm of thickness along the rostro-caudal extension of the ischemic cortical region. For Dkk3 expression analysis, slices were incubated overnight with a goat polyclonal anti-Dkk3 antibody (1:50, RD System) and then for 1 h at room temperature (RT) with a secondary biotinylated anti-goat IgG (Vector Laboratories, Burlingame, CA, USA). 3,3-Diaminobenzidine tetrachloride (Sigma-Aldrich, Milan, Italy) was used as chromogen substrate for detection of the immunoreactivity.

For double immunofluorescence analysis slices were incubated overnight at +4°C with a mixture containing a goat polyclonal anti-Dkk3 antibody (1:50, RD System) and a mouse monoclonal anti-glial fibrillary acid protein (GFAP, 1:300, Sigma-Aldrich). Following incubation with the primary antibodies, slices were incubated for 1 h at RT with a mixture containing the Cy3-conjugated anti-goat Cy3 IgG (1:300, Chemicon, Temecula, CA, USA) antibody and the fluorescein-conjugated anti-mouse IgG (1:50, Vector Laboratories).

### Cultures of Mouse Cortical Astrocytes

Pure primary cultures of cortical astrocytes were prepared from CD1 mice, or from Dkk3^−/−^ mice or their wild-type counterparts 1–3 days after birth as previously described (Rose et al., 1992). Briefly, cerebral cortices were dissected, digested with 0.25% trypsin for 5 min and then centrifuged at 1,000 *g* for 7 min.

After centrifugation, pellets were resuspended in 1 ml of the plating medium (PM; MEM-Eagle’s salts supplemented with 10% heat-inactivated fetal bovine serum, 10% heat-inactivated horse serum, 2 mM glutamine, 100 U/ml penicillin, 100 μg/ml streptomycin 25 mM sodium bicarbonate and 21 mM glucose), subjected to a gentle trituration with a large bore pipette until a cloudy suspension is obtained. After that, the suspensions were combined with appropriate amount of PM (2 hemispheres in 10 ml of PM) and plated onto 100 × 20 mm dishes or 24-well plates (Falcon Primaria, Lincoln Park, NJ, USA; 2 hemispheres/plate). Cultures were grown at 37°C in a humidified CO_2_ atmosphere until confluence was obtained (10–14 days *in vitro*—DIV). For cell viability studies (see below) we used the following experimental conditions: (i) astrocytes prepared from wild-type or Dkk3^−/−^ mice were exposed to high concentrations of H_2_O_2_ (250 μM, 1 h) or to 4 h of glucose deprivation; (ii) astrocyte prepared from CD1 mice were exposed to H_2_O_2_ (100 μM, 1 h) in the absence or presence of hrDkk3 (10 ng/ml). Cell lysates were also used for Western blot analysis of Dkk3 and VEGF protein levels.

### Mixed Cortical Cells

Mixed cortical cell cultures containing both neurons and astrocytes were prepared from fetal mice at 14–16 days of gestation. Cerebral cortices were dissected and centrifuged at 1,000 *g* for 7 min. The pellets were resuspended in 1 ml of PM and subjected to a gentle trituration. The obtained homogenates were combined with appropriate amount of PM (four hemispheres in 10 ml of PM) for a final cell density of approximately 2.5 × 10^5^ cells in each well. Cortical cells were plated in 24-well plates (Falcon Primaria) on a layer of confluent astrocytes (14 DIV), using a PM of MEM Eagle’s salts supplemented with 10% heat-inactivated horse serum, 10% fetal bovine serum, glutamine (2 mM), 100 U/ml penicillin, 100 μg/ml streptomycin and glucose (21 mM). After 3–5 days *in vitro*, non-neuronal cells division was halted by 1–3 days exposure to 2 μM cytosine arabinoside. Only mature cultures (13–14 DIV) were used for experiments. Mouse mixed cortical cultures prepared from Dkk3^−/−^ or their wild-type littermates were challenged for 10 min with NMDA (100 μM) at RT in HEPES-buffered salt solution containing (in mM): NaCl, 120; KCl, 5.4; MgCl_2_, 0.8; CaCl_2_, 1.8; HEPES, 20; and glucose, 15. Afterwards, cultures were incubated at 37°C for the following 20 h in MEM-Eagle’s supplemented with 15.8 mM NaHCO_3_ and 25 mM glucose.

Mixed cortical cultures prepared from CD1 mice were also challenged for 10 min with NMDA (45, 80 or 150 μM) and incubated for the following 20 h in the absence or presence of hrDkk3 (10 ng/ml) combined or not with the type-2 VEGF receptor (VEGFR2) inhibitor, ZM3238881 (10 μM) or with hrVEGF (0.1 or 1 ng/ml).

### Assessment of Cell Viability in Cultured Astrocytes

Flow cytometry analysis (FACS) was performed for the assessment of cell viability using the FITC Annexin V Apoptosis Detection kit (BD Pharmingen, San Diego, CA, USA). Cell culture media were collected, and cells were harvested by incubation with 1 ml of 0.25% trypsin/EDTA (Lonza, Milan, Italy) for 5 min at 37°C. Trypsinization was stopped by addition of complete medium and the suspension was centrifuged at 1,200 rpm for 5 min at RT. Each pellet was washed with cold phosphate buffered saline. Then, tubes were vortexed thoroughly and centrifuged again as above. Cells were gently resuspended in 200 μl of binding buffer. Then, cell suspension was added to 5 μl of Annexin V-FITC and 10 μl of PI. Samples were mixed and incubated in the dark at 4°C for 20 min. Ten thousand cells were analyzed by FACS on a BD Accuri C6 flow cytometer to count live cells. All AnV/PI measurements were performed in duplicate, after setting the discriminator to exclude debris by forward and right angle scatter. For this assay, red fluorescence was measured corresponding to the red color of propidium iodide (FL-3 detector), and green fluorescence was measured corresponding to the green color of ANX-V (FL-1 detector). Both negative control (untreated cells) and positive control (cells treated with a high dose of H_2_O_2_) were included in the analysis.

### Assessment of NMDA-Induced Neuronal Death in Mixed Cortical Cultures

Neuronal death was assessed by measuring the activity of lactate dehydrogenase (LDH) released into the medium using a commercial kit (Roche Diagnostic GmbH, Mannheim, Germany), 20 h after the NMDA pulse.

### Western Blot Analysis

Western blot analysis from lysates of astrocyte cell cultures or whole brains was performed using the following antibodies: goat polyclonal anti-Dkk3 (RD System); rabbit polyclonal anti-VEGF antibody (1:1,000, Abcam, Cambridge, UK; code: ab46154), rabbit polyclonal anti-phospho-AKT (Ser473; 1:1,000, Cell Signaling Technology, Danvers, MA, USA, code: 9271), rabbit polyclonal anti-AKT (1:1,000, Cell Signaling Technology, code: 9272), rabbit polyclonal anti-Bcl2 (1:200, Santa Cruz Biotechnology, Dallas, TX, USA, code: sc-492) and mouse monoclonal anti-β-actin antibody (1:50,000, Sigma-Aldrich, code: A5441). After incubation with primary antibodies, filters were washed and incubated with secondary peroxidase-coupled anti goat (1:2,000, Calbiochem, Milan, Italy, code: 401515), anti-rabbit (1:6,000, Calbiochem, code: 401393) or anti-mouse (1:5,000, Santa Cruz Biotechnology, code: sc-2005) antibodies. Immunostaining was revealed by enhanced chemiluminescence (GE Healthcare, Milan, Italy).

### Statistical Analyses

**Figure 1:**
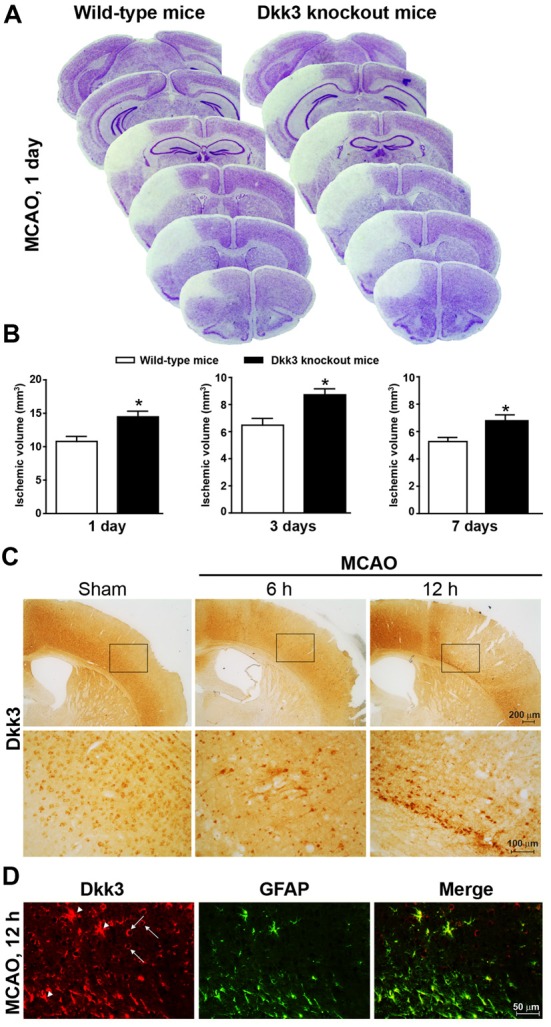
Dickkopf-3 (Dkk3) is protective against ischemic neuronal death. **(A)** Nissl staining in 10 μm coronal mouse brain sections collected every 320 μm along the rostro-caudal extension of the ischemic lesion in wild-type and Dkk3 knockout mice 1 day after middle cerebral artery occlusion (MCAO). **(B)** Infarct size in wild-type and Dkk3 knockout mice 1 day, 3 days or 7 days following MCAO. Values are means + SEM. *p < 0.05 vs. wild-type mice (Student’s *t*-test; day 1: *n* = 7–9, *p* = 0.0073, *t*_(14)_ = 3.135; day 3: *n* = 4–5, *p* = 0.0143, *t*_(7)_ = 3.24; day 7: *n* = 4–7, *p* = 0.0387, *t*_(9)_ = 2.42). **(C)** Immunohistochemical analysis of Dkk3 in the cerebral cortex of wild-type mice at 6 h and 12 h following MCAO. Low and high magnification images are shown. **(D)** Double fluorescence immunostaining of Dkk3 and GFAP in the ischemic cortex of wild-type mice 12 h following MCAO. Arrowheads show Dkk3 expression in astrocytes. Arrows show Dkk3 expression in GFAP-negative cells.

**Figure 2:**
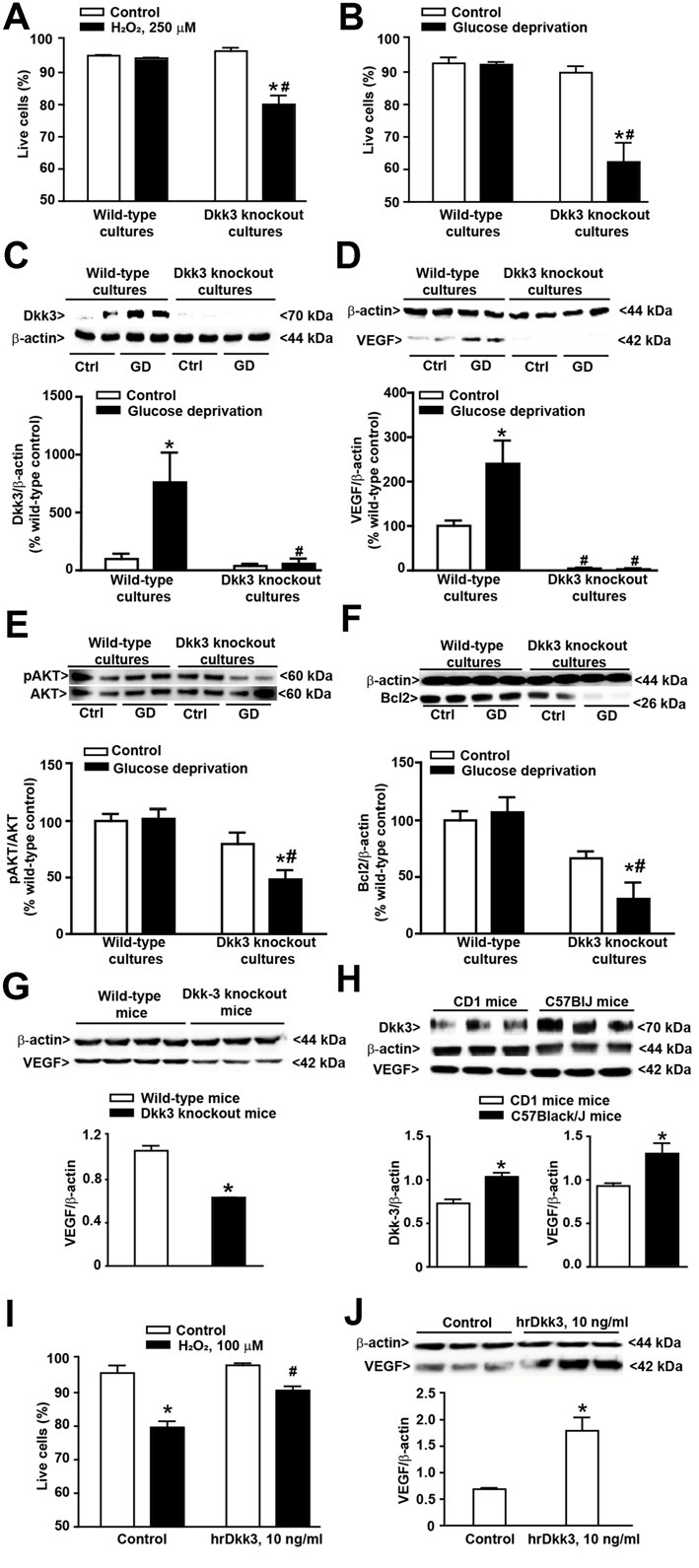
Protective activity of Dkk3 in cultured astrocytes exposed to oxidative damage or glucose deprivation. **(A)** Flow cytometry analysis (FACS) of live cells in cultured cortical astrocytes from wild-type and Dkk3 knockout mice (C57BL/6J strain) following 1 h of incubation with H_2_O_2_ (250 μM). Values are means + SEM (*n* = 3–4 per group). *p* < 0.05 vs. the respective control cultures not exposed to H_2_O_2_ (*), or vs. wild-type cultures exposed to H_2_O_2_ (^#^two-way ANOVA + Fisher’s least significant difference (LSD); genotype, *p* < 0.001, *F*_(1,11)_ = 15.79; treatment, *p* < 0.001, *F*_(1,11)_ = 28.24; genotype × treatment, *F*_(1,11)_ = 23.43). **(B)** Same as in **(A)** but after exposure to 4 h of glucose deprivation. Values are means + SEM. (*n* = 6–9 per group). *p* < 0.05 vs. the respective cultures not exposed to glucose deprivation (*) or vs. wild-type cultures exposed to glucose deprivation (^#^two-way ANOVA + Fisher’s LSD; genotype, *p* < 0.001, *F*_(1,25)_ = 26.73; treatment, *p* < 0.001, *F*_(1,25)_ = 19.33; genotype × treatment, *F*_(1,25)_ = 18.26). Dkk3 protein levels in cortical cultures of astrocytes after 4 h of glucose deprivation are shown in **(C)**. Values are means + SEM (*n* = 4 per group). *p* < 0.05 vs. the respective cultures not exposed to glucose deprivation (*) or vs. wild-type cultures exposed to glucose deprivation (^#^two-way ANOVA + Fisher’s LSD; genotype, *p* = 0.003, *F*_(1,12)_ = 8.18; treatment, *p* = 0.004, *F*_(1,12)_ = 6.53; genotype × treatment, *F*_(1,12)_ = 5.82). VEGF protein levels in cortical cultures of astrocytes after 4 h of glucose deprivation are shown in **(D)**. Values are means + SEM (*n* = 4 per group). *p* < 0.05 vs. the respective cultures not exposed to glucose deprivation (*) or vs. wild-type cultures exposed to glucose deprivation (^#^two-way ANOVA + Fisher’s LSD; genotype, *p* < 0.001, *F*_(1,12)_ = 38.54; treatment, *p* = 0.003, *F*_(1,12)_ = 6.60; genotype × treatment, *F*_(1,12)_ = 6.90). Phospho-AKT protein levels in cortical cultures of astrocytes after 4 h of glucose deprivation are shown in **(E)**. Values are means + SEM (*n* = 6 per group). *p* < 0.05 vs. the respective cultures not exposed to glucose deprivation (*) or vs. wild-type cultures exposed to glucose deprivation (^#^two-way ANOVA + Fisher’s LSD; genotype, *p* < 0.001, *F*_(1,20)_ = 19.40; treatment, *p* = 0.016, *F*_(1,20)_ = 3.09; genotype × treatment, *F*_(1,20)_ = 3.93). Bcl2 protein levels in cortical cultures of astrocytes after 4 h of glucose deprivation are shown in **(F)**. Values are means + SEM (*n* = 4 per group). *p* < 0.05 vs. the respective cultures not exposed to glucose deprivation (*) or vs. wild-type cultures exposed to glucose deprivation (^#^two-way ANOVA + Fisher’s LSD; genotype, *p* < 0.001, *F*_(1,12)_ = 25.46; treatment, *p* = 0.038, *F*_(1,12)_ = 1.74; genotype × treatment, *F*_(1,12)_ = 3.93). VEGF protein levels in the whole brain of wild-type and Dkk3 knockout mice are shown in **(G)**. Values are means + SEM (*n* = 3–4 per group). *p < 0.05 vs. wild-type mice (Student’s *t-test*; *p* = 0.0005, *t*_5_ = 7.98). Dkk3 and VEGF protein levels in the whole brain of CD1 and C57BL/6J mice are shown in **(H)**. Values are means ± SEM (*n* = 3 per group). *p < 0.05 vs. CD1 mice (Student’s *t-test*; Dkk3, *p* = 0.0099, *t*_(4)_ = 4.62; VEGF, *p* = 0.0384, t_(4)_ = 3.04). **(I)** FACS analysis of live cells in cultures of cortical astrocytes from CD1 mice incubated for 12 h with hrDkk3 (10 ng/ml) prior to a 1 h-exposure to H_2_O_2_ (100 μM). Values are means + SEM (*n* = 3 per group). *p* < 0.05 vs. the respective control cultures not exposed to H_2_O_2_ (*p = 0.0001), or vs. the respective cultures not treated with hrDkk3 (^#^*p* = 0.0013; one-way ANOVA + Fisher’s LSD; *F*_(3,8)_ = 26.2). VEGF protein levels in lysates of cultured astrocytes from CD1 mice treated with hrDkk3 (10 ng/ml) for 12 h are shown in **(J)**. Values are means ± SEM (*n* = 3 per group). *p < 0.05 vs. controls (Student’s *t-test*; *p* = 0.0119, t_(4)_ = 4.38). Ctrl = untreated controls.

**Figure 3:**
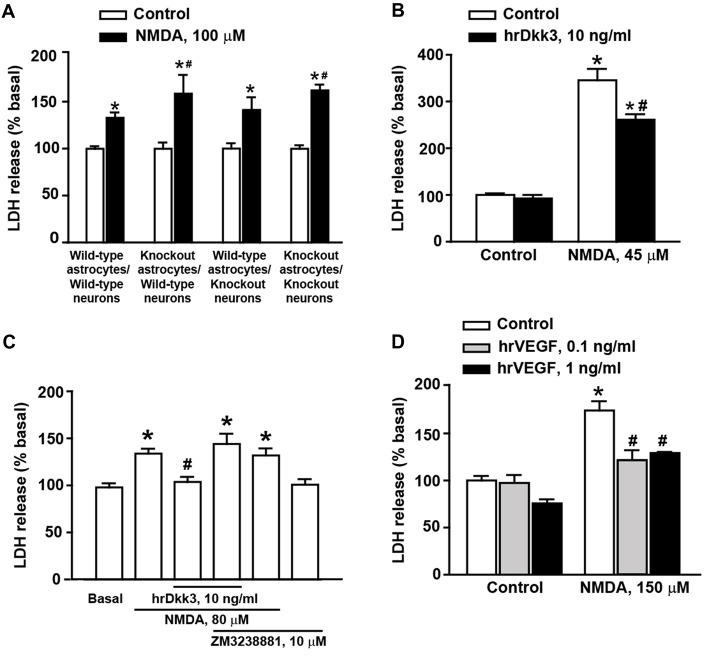
Dkk3 is protective against excitotoxic neuronal death in mixed cortical cultures. Neuronal death in mixed cortical cultures prepared from different mouse genotypes (all C57BL/6J strain) and challenged with 100 μM *N*-methyl-D-aspartate (NMDA) is shown in **(A)**. Values are means + SEM (*n* = 4–8 per group). *p* < 0.05 vs. the respective cultures not treated with NMDA (*), or vs. cultures treated with NMDA from wild-type mice in both neurons astrocytes and neurons (^#^two-way ANOVA + Fisher’s LSD; genotype, *p* = 0.002 for astrocytes knockout/neurons knockout; *p* = 0.026 for astrocytes knockout/neurons wild-type, *F*_(3,40)_ = 2.06; treatment, *p* < 0.001 for astrocytes wild-type/neurons wild-type, astrocytes knockout/neurons wild-type and astrocytes knockout/neurons knockout; *p* = 0.003 for astrocytes wild-type/neurons knockout, *F*_(1,40)_ = 77.52; genotype × treatment, *F*_(3,40)_ = 2.07). Neuronal death in cultures from CD1 mice challenged with 45 μM NMDA followed or not by 20-h exposure to hrDkk3 (10 ng/ml) is shown in **(B)**. Values are means + SEM (*n* = 4 per group). *p* < 0.05 vs. cultures not treated with NMDA (*p < 0.0001) or vs. cultures challenged with NMDA not exposed to hrDkk3 (*p* = 0.0009; one-way ANOVA + Fisher’s LSD; *F*_(3,12)_ = 83.69). Data obtained in cultures from CD1 mice challenged with 80 μM NMDA followed or not by 20-h exposure to hrDkk3 (10 ng/ml) w/wo ZM3238881 (10 μM) is shown in **(C)**. Values are means + SEM (*n* = 5–8 per group). *p* < 0.05 vs. cultures not treated with NMDA (*p = 0.0005 for NMDA, *p* < 0.0001 for NMDA+ hrDkk3 + ZM323881, *p* = 0.0012 for NMDA+ ZM323881) or vs. cultures challenged with NMDA and not exposed to hrDkk3 (^#^*p* = 0.0045; one-way ANOVA + Fisher’s LSD; *F*_(5,32)_ = 8.39). Neuroprotection by hrVEGF against NMDA toxicity (here, 150 μM) in mixed cortical cultures from CD1 mice is shown in **(D)**. Cells were exposed to hrVEGF for 20 h following the NMDA pulse. Values are means + SEM (*n* = 4–11 per group). *p* < 0.05 vs. cultures not treated with NMDA (*p < 0.0001) or vs. cultures challenged with NMDA not exposed to hrVEGF (^#^*p* < 0.0001; one-way ANOVA + Fisher’s LSD; *F*_(5,31)_ = 22.26).

Statistical analyses were performed as follows: (i) Unpaired *t*-test (Figures 1B, 2E,F,H); (ii) One-way ANOVA followed by Fisher’s Least Significant Difference (LSD; Figures 2G, 3B–D); and, (iii) Two-way ANOVA followed by Fisher’s LSD (Figures 2A–D, 3A). Statistical significance was set at a *p* value < 0.05.

## Results

### Increased Infarct Size in Ischemic Mice Lacking Dkk3

To examine whether endogenous Dkk3 shapes brain vulnerability to ischemic damage, we used mice subjected to permanent MCA occlusion (MCAO, see section “Materials and Methods”). Ischemic infarct, detected by histological analysis, involved the mid-lateral portion of the cerebral cortex ipsilateral to MCAO (Figures 1A,B). We measured infarct volume at three time points (1, 3 and 7 days following MCAO) in which ischemic infarct is fully developed under our experimental conditions (Mastroiacovo et al., 2009). Infarct volume was significantly greater in Dkk3^−/−^ mice at all selected time points (Figures 1A,B), suggesting that endogenous Dkk3 restrains brain vulnerability to ischemic damage. Expression of Dkk3 was assessed by immunohistochemistry at 6 and 12 h post-ischemia, when ischemic damage is partial and near-to-maximal, respectively (Mastroiacovo et al., 2009). An increase in Dkk3 immunoreactivity was detected in the medial and inferior portion of the cerebral cortex surrounding the ischemic focus, a region that likely incorporates the ischemic penumbra, at both time points. However, the increase was more substantial at 12 h following MCAO (Figure 1C). Double fluorescent staining for GFAP and Dkk3 at this time point indicated that astrocytes largely contributed to the increased expression of Dkk3 at the borders of the ischemic focus (Figure 1D), although other cell types also expressed Dkk3 (see arrowheads in Figure 1D). This evidence raised the possibility that induction of Dkk3 in astrocytes could represent a defensive mechanism against ischemic neuronal death. To explore this mechanism in further detail, we examined the protective activity of Dkk3 in primary cultures of astrocytes and in mixed cortical cultures containing both astrocytes and neurons.

### Dkk3 Supports Cell Viability in Primary Cultures of Cortical Astrocytes

We first used cultures of cortical astrocytes prepared from wild-type and Dkk3^−/−^ mice (C57BL/6J strain). Interestingly, cultures from wild-type mice were highly resistant to oxidative stress induced by high concentrations of H_2_O_2_ (250 μM). In contrast, these concentrations of H_2_O_2_ significantly reduced cell viability in cultures prepared from Dkk3^−/−^ mice (Figure 2A). Similar findings were obtained after 4 h of glucose deprivation, which were not harmful in cultures from wild-type mice but substantially reduced cell survival in cultures from Dkk3^−/−^ mice (Figure 2B). In cultures from wild-type mice, glucose deprivation substantially increased Dkk3 protein levels (Figure 2C). Knowing that Dkk3 induces VEGF expression in endothelial cells (Busceti et al., 2017), we also measured VEGF protein levels in response to glucose deprivation. Similarly to Dkk3, VEGF protein levels increased after 4 h of glucose deprivation. Remarkably, VEGF levels were very low and did not increase after glucose deprivation in cultures from Dkk3^−/−^ mice (Figure 2D). We extended the analysis to molecules that lie downstream of VEGF receptor activation and likely contribute to cell protection. VEGF receptor activation is known to stimulate the phosphatidylinositol-3-kinase (PI3K) pathway, which has a widespread role in cell protection mechanisms. Interestingly, glucose deprivation in astrocytes lacking Dkk3 caused a substantial reduction in phospho-Akt levels, which was not observed in wild-type astrocytes (Figure 2E). Activation of the PI3K pathway induces the expression of the antiapoptotic gene, Bcl-2 (Pugazhenthi et al., 2000). We found a significant reduction in Bcl-2 protein levels in response to glucose deprivation in astrocytes lacking Dkk3, but not in wild-type astrocytes (Figure 2F). Taken together, data obtained in cultured astrocytes suggest that Dkk3 is cytoprotective via the induction of VEGF and the resulting activation of the PI3K/Bcl-2 axis. To examine whether VEGF was under the control of Dkk3 also in intact animals, we measured VEGF protein levels in the whole brain of wild-type and Dkk3^−/−^ mice. Mice lacking Dkk3 showed a substantial reduction of VEGF levels in whole brain lysates (Figure 2G).

In the initial stage of our study of Dkk3 in mice we were intrigued from the findings that Dkk3 levels were lower in the whole brain of CD1 mice with respect to the whole brain of age-matched C57BL/6J mice (Figure 2H). VEGF protein levels were also lower in the whole brain of CD1 mice (Figure 2H), further supporting the hypothesis that VEGF is under the control of Dkk3. Moving from this finding, we wondered whether astrocyte cultures prepared from CD1 mice could be more sensitive to a cytotoxic insult. We addressed this question by challenging cultures with 100 μM H_2_O_2_, and found that these concentrations of H_2_O_2_, which were lower than those used in cultures from C57BL/6J mice, were toxic to CD1 mouse astrocytes (Figure 2I). Using these cultures, we could also demonstrate that exogenous application of hrDkk3 (10 ng/ml) was protective (Figure 2I). Interestingly, hrDkk3 also enhanced VEGF protein levels by about 2.5 fold in cultured astrocytes from CD1 mice (Figure 2J).

### The Dkk3/VEGF Axis Is Protective Against Excitotoxic Neuronal Death in Mixed Cortical Cultures

To examine whether endogenous Dkk3 was protective against excitotoxic neuronal death, we used cultures prepared from wild-type or Dkk3^−/−^ mice challenged for 10 min with 100 μM NMDA. Neuronal death was assessed 20 h after the NMDA pulse. In control cultures, the extent of NMDA toxicity was limited perhaps owing of the C57BL/6J strain of these mice (Figure 3A).

NMDA toxicity was amplified when cultures contained astrocytes derived from Dkk3^−/−^ mice (Figure 3A). In contrast, toxicity was unchanged when neurons from Dkk3^−/−^ mice were plated over a monolayer of astrocytes from wild-type mice (Figure 3A). These findings suggested that Dkk3 produced from astrocytes acted on neighbor neurons to attenuate excitotoxic death.

We next used cultures prepared from CD1 mice, which, in general, were more sensitive to NMDA toxicity. However, the extent of neuronal death varied in different culture preparations, and the appropriate concentrations of NMDA were established from time to time on the basis of data obtained with concentration-response curves of NMDA in sister cultures performed the day before the experiment. Addition of hrDkk3 was neuroprotective when applied to the cultures immediately after a pulse with 45 μM NMDA, and maintained in the medium for the following 20 h (Figure 3B). To examine whether VEGF was involved in Dkk3-induced neuroprotection, cultures from CD1 mice were exposed to hrDkk3 in the absence or presence of the type-2 VEGF receptor (VEGFR2) inhibitor, ZM3238881 (10 μM; Whittles et al., 2002). Pharmacological blockade of VEGFR2 abrogated the neuroprotective effect of hrDkk3 against NMDA toxicity (here, 80 μM; Figure 3C). In another experiment, we could also demonstrate that application of hrVEGF (0.1 or 1 ng/ml) was protective against NMDA toxicity (150 μM) in mixed cortical cultures (Figure 3D).

## Discussion

We have shown here that: (i) endogenous Dkk3 limits ischemic brain damage in mice and acts as a protective molecule in cultured neurons and astrocytes; and (ii) this action is not saturated and can be further enhanced by exogenous Dkk3. One interesting observation was that Dkk3 levels in CD1 and C57BL/6J mice were inversely related to the vulnerability of the respective cultured astrocytes or neurons to oxidative or excitotoxic damage. This suggests that endogenous Dkk3 shapes the responsiveness of CNS cells to environmental insults.

Activation of the Wnt pathway is known to support neuronal survival, and induction of the Wnt inhibitor, Dkk-1, contributes to neuronal death in experimental animal models of cerebrovascular disorders (Cappuccio et al., 2005; Zhang et al., 2008; Mastroiacovo et al., 2009; He et al., 2016; Li et al., 2017), AD (Caricasole et al., 2004; Wang et al., 2016), temporal lobe epilepsy (Busceti et al., 2007), and stress-related disorders (Matrisciano et al., 2011). Dkk3 differs from all other Dkk family members because its modulatory action on Wnt signaling is complex and context-dependent. In recombinant cells, Dkk3 inhibits β-catenin/TCF nuclear signaling stimulated by Wnt7a, but only when the Wnt co-receptor, LRP6 is overexpressed (Caricasole et al., 2003). In contrast, Dkk3 positively modulates the canonical Wnt signaling pathway in the heart and kidney (Federico et al., 2016; Lu et al., 2016; Gröne et al., 2017).

Our finding that Dkk3 induced VEGF expression in human vascular endothelial cells (Busceti et al., 2017) added a new player to the complex scenario of the mechanism of action of Dkk3.

**Figure 4:**
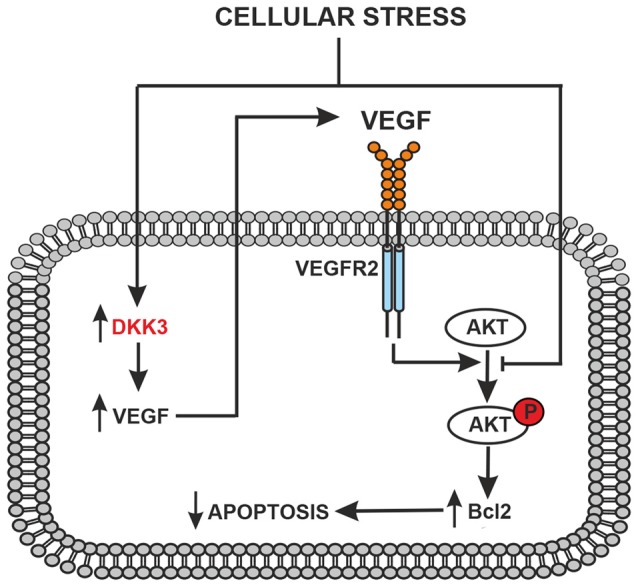
Schematic representation of the protective feedback Dkk3-mediated in astrocytes and/or neurons exposed to cellular stress (glucose deprivation, oxidative stress or ischemia). Dkk3 leads to VEGF upregulation with ensuing VEGFR2 activation, AKT phosphorylation and upregulation of the antiapoptotic factor Bcl2.

The following observations suggest that a Dkk3/VEGF axis is protective in cultured astrocytes and neurons: (i) VEGF were very low under basal conditions and were not induced by glucose deprivation in astrocytes lacking Dkk3; (ii) two protective mechanisms that lie downstream of VEGFR2, i.e., activation of the PI3K pathway and the resulting induction of Bcl-2, were down-regulated in astrocytes lacking Dkk3; (iii) astrocyte cultures prepared from CD1 mice, which showed lower brain levels of Dkk3 and VEGF, were more sensitive to toxic insults; (iv) hrDKK3 was protective in cultured astrocytes and induced VEGF; (v) pharmacological blockade of VEGFR2 abrogated the neuroprotective activity of hrDkk3 in mixed cultures; and (vi) hrVEGF itself was neuroprotective in mixed cultures. On the basis of these findings, we hypothesized the following scenario that could be targeted by therapeutic intervention: in response to cellular stressors Dkk3 produced by either astrocytes or neurons induces the expression of VEGF, which, by activating VEGFR2, causes cytoprotection through the PI3K pathway or via other mechanisms (Figure 4).

While ischemic infarct was greater in mice lacking Dkk3, we have no evidence so far that endogenous Dkk3 is protective against ischemic neuronal damage by a mechanism mediated by VEGF. The role of VEGF in CNS disorders is complex and not homogenous, as highlighted in a recent review (Shim and Madsen, 2018). Excessive VEGF might have detrimental effects in cerebrovascular disorders and age-related neurodegenerative disorders by disrupting endothelial barriers, thereby inducing oedema and inflammation. On the other side of the coin, VEGF has shown neuroprotective activity in models of AD, Parkinson’s disease and stroke (reviewed by Shim and Madsen, 2018; see also Introduction and references therein). It will be interesting to investigate whether exogenous VEGF rescues the pro-ischemic phenotype of Dkk3 mice. However, this task is difficult to achieve because of the pleiotropic effects of VEGF in CNS cells, which require the analysis of different doses of VEGF delivered in different territories of the ischemic damage in different time windows.

The dual effect of VEGF limits the use of either pro- or anti-VEGF strategies (e.g., anti-VEGF monoclonal antibodies/small molecule VEGFR inhibitors and mesenchymal stem cells engineered to produce VEGF_A_, respectively) in the treatment of CNS disorders (reviewed by Shim and Madsen, 2018). Perhaps strategies aimed at enhancing Dkk3 levels might help to overcome these limitations by optimizing the amount of VEGF that is endogenously produced under pathological conditions.

As opposed to other members of the Dkk family (see Zorn, 2001), the identity of the receptor(s) that mediate(s) the biological action of Dkk3 is unknown. We have shown that hrDkk3 induces VEGF by activating the activin-like kinase-1 (ALK-1) TGF-β receptor subtype in human endothelial cells. In these cells, Dkk3 increased Smad1/5/8 phosphorylation, thereby activating VEGF gene transcription (Busceti et al., 2017). Whether Dkk3 directly activates TGF-β receptors or interacts with an unknown receptor that positively modulates TGF-β receptors is unknown. Dkk3 can also induce VEGF by activating the Wnt/β-catenin pathway, as shown in human retinal pigment epithelial cells (Wang et al., 2016). Which of these (or other) mechanisms mediates the induction of VEGF by Dkk3 in astrocytes remains to be determined.

In conclusion, we offered the first evidence that Dkk3 protects cultured neurons and astrocytes through a mechanism that involves the induction of VEGF. A limitation of the study is that we specifically focused on the involvement of VEGF in the action of Dkk3 and other potential mechanisms have been neglected.

In both *in vitro* and *in vivo* models, endogenous Dkk3 behaved as a defensive molecule, raising the possibility that strategies aimed at enhancing Dkk3 production are protective. The analysis of how neurotransmitter receptors expressed by astrocytes control Dkk3 gene expression might be a starting point in the identification of these strategies.

## Author Contributions

CB performed *in vitro* experiments of glucose deprivation, histological and immunohistochemical analyses, statistical analyses, designed experiments and wrote the manuscript. LD prepared cell cultures and performed experiments of NMDA-induced excitotoxicity. FB performed *in vitro* experiments of oxidative stress in cultured astrocytes and western blot analysis. CN provided Dkk3^−/−^ mice. FM and GBo performed *in vivo* experiments using the model of permanent focal ischemia. PD and AT contributed to cell culture experiments and developed the colony of Dkk3^−/−^ mice. GBa, VB, FF, MV, SR and FN designed experiments, supervised research and contributed to writing the manuscript.

## Conflict of Interest Statement

The authors declare that the research was conducted in the absence of any commercial or financial relationships that could be construed as a potential conflict of interest.
